# New Appliances and Things Medical

**Published:** 1898-05-21

**Authors:** 


### NEW APPLIANCES AND THINGS MEDICAL.

[We shall be glad to receive, at our Office, 28 & 29, Southampton Street, Strand, London, W.C., from the manufacturers, specimens of all new preparations and appliances which may be brought out from time to time.]

### BYNO-PHOSPHATES.

(ALLEN AND HANBURYS, PLOUGH COURT, LOMBARD STREET, E.C.)

The advantages of phosphates when used as a tonic for delicate and growing children are now too generally known to require notice. That special preparation of these salts, known as Byno-phosphate, which is under review, is one of the most elegant on the market; it is, in fact, an improved form of the well-known Parrish's Food, combined with bynin, a most satisfactory form of malt extract. In byno-phosphate is combined the valuable properties of iron phosphate, together with the nutritive and digestive power of malt.

### BARFF BORO-GLYCERIDE.

(THE KREOCHYLE COMPANY, VIADUCT HOUSE, FARRINGDON STREET, E.C.)

We have received from the Kreochyle Company two samples of "Barff Boro-glyceride," one in a hydrated form and the other sample as a transparent or glacial form of preparation. Boro-glyceride is a chemical compound which has been produced by the interaction of boracic acid and glycerine at a high temperature, forming what is chemically known as glyceryl borate, and therefore is not a mere mechanical mixture of boracic acid and glycerine. Boro-glyceride is well-known as a food preservative and as an antiseptic in operative surgery. It has the advantage of being more readily soluble in water than boracic acid, and is recommended as being a more powerful antiseptic.

